# A Combat Journey With Pulmonary Rehabilitation and Palliative Care in a Patient With Pleural Effusion, Secondary to Metaplastic Breast Carcinoma

**DOI:** 10.7759/cureus.30545

**Published:** 2022-10-21

**Authors:** Nikita Kaple, Moli Jain, Vaishnavi Yadav, Pallavi Bhakaney

**Affiliations:** 1 Department of Physiotherapy, Ravi Nair Physiotherapy College, Datta Meghe Institute of Medical Sciences, Wardha, IND; 2 Department of Cardiorespiratory Physiotherapy, Ravi Nair Physiotherapy College, Datta Meghe Institute of Medical Sciences, Wardha, IND; 3 Department of Cardiorespiratory Physiotherapy, Resident, Ravi Nair Physiotherapy College, Datta Meghe Institute of Medical Sciences, Wardha, IND

**Keywords:** breast cancer, metaplastic, activity pacing strategies, functional independence, pulmonary rehabilitation, thoracentesis, palliative care, malignant pleural effusion, metaplastic breast cancer

## Abstract

Metaplastic breast cancer is an uncommon and fatal condition. It is described histologically as a tumor having epithelial differentiation into squamous and mesenchymal components, with numerous elements frequently co-existing in the same tumor. This case study sought to provide analgesic treatment in a case with malignant pleural effusion related to breast cancer based on evidence. A 67-year-old female with a known history of metaplastic breast carcinoma came to the tertiary care hospital with complaints of breathlessness which progressed to grade II on the Modified Medical Research Council (mMRC) Dyspnoea Scale, a cough with mucoid expectoration, restlessness, nausea, and reduced appetite for eight days. Diagnostic findings revealed bilateral pleural effusion (left>right). Laboratory investigations revealed that the excess fluid accumulated was transudate in nature, according to the Light’s criteria. This case report illustrates the strategy, management, and importance of adherence to pulmonary rehabilitation and painkiller care physiotherapy for patients with metaplastic breast cancer and pleural effusion to achieve the best possible physical and mental health.

## Introduction

Metaplastic breast cancer patient care entails close cooperation between oncology and palliative care providers to treat the numerous symptoms and reduced quality of life that are a result of this disease [1]. It is a rare kind of cancer that affects around 1% of all women and one in every four women. Since this is an invasive cancer, it has the potential to spread to other bodily areas [2]. According to an estimated 2.3 million new cases [3], breast cancer will account for one out of every eight malignancies identified in 20. Chemotherapy has revolutionized cancer survival by enhancing survival and quality of life, but it also has several unintended consequences. The lung is the most commonly affected organ in chemotherapy-related problems due to drug toxicity or infections caused by immunosuppression, with immune-mediated harm less prevalent [4]. Pleural effusion, which can be malignant, is one of the most common consequences. It is confined by the existence of cancerous cells in the pleural fluid or tissue [5]. The presence of cancerous cells in the pleural fluid or tissue serves as a telltale sign.

Anesthetic maintenance repair matches the World Health Organization's proposed bio-psycho-social approach to health care by meeting patients' physical, psychological, social, and spiritual requirements. In addition to symptom management and therapy, this essential medical service also addresses effect reduction in patients with chronic, irreversible disorders. It must be used as required to enhance and maintain the quality of life of patients. Physiotherapy aims to maximize movement and function, which is necessary for maximal health when mobility and function are diminished by age, accident, or disease [6]. It includes cancer, HIV, neurological illnesses, cardiac problems, and mental illness. Physiotherapy helps improve the quality of life and well-being of such patients. It includes medication, nutritional modifications, relaxation techniques, and emotional support. Based on the evidence [7], the goal of this case study was to offer anodyne treatment to patients with malignant pleural effusion associated with breast cancer.

## Case presentation

**Figure 1:**
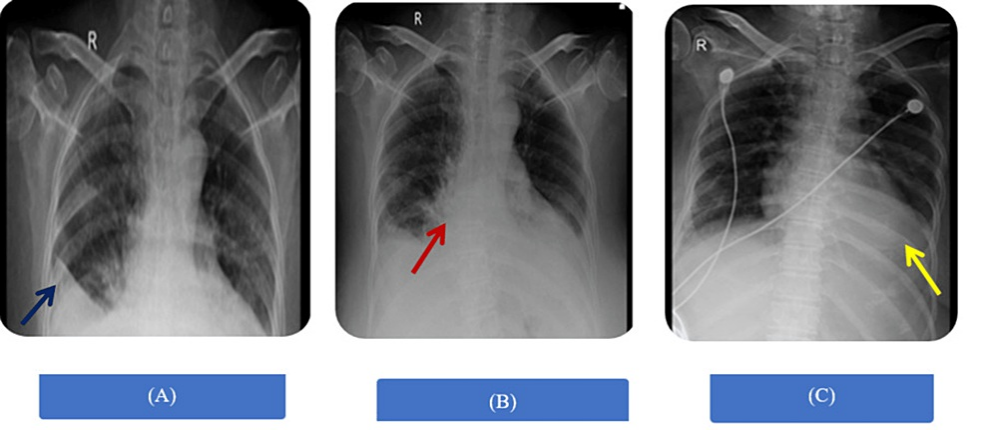
The patient's chest X-ray showed: (A) Homogenous opacities seen on bilateral lower zones with obliterated costophrenic angle on the right side; (B) Homogenous opacities seen on the right side with the presence of air bronchogram; (C) Obliteration of left costophrenic angle with diffusely visible cardiac margins on the ipsilateral side.

A 67-year-old female with a known history of left metaplastic breast carcinoma presented to our tertiary care hospital with complaints of a change in breast size, a lump or thickening of the skin to the nipple, pain in the breast, gradual breathlessness with a grade II Modified Medical Research Council (mMRC) Dyspnea Scale, a cough with mucoid expectoration, restlessness, nausea, and decreased appetite. These complaints had been present for eight days. Related concerns were that she was having trouble executing her regular chores and was tired, which bothered her. History and medical records revealed that she took ten cycles of chemotherapy with paclitaxel and had a documented history of right-sided pleural effusion, for which thoracocentesis was performed in May 2021. She also had diabetes mellitus and gave a record of the ingestion of one tablet of Gluconom SR daily. After being admitted, she received several radiological and laboratory tests, including mammography, high-resolution computed tomography (HRCT), and the chest X-ray noted in Figure 1. According to Light's criteria, laboratory tests showed that the extra fluid accumulated was transudate in character. Diagnostic findings indicated bilateral pleural effusion (left>right). Further management included a thoracocentesis procedure to aspirate the excess pleural fluid. The patient was started with an injection and kept under close monitoring in the Intensive Care Unit (ICU). Given the symptoms of exhaustion, restlessness, and lack of breath, physiotherapy was recommended and supplied directly. Table 1 shows the events that occurred during the plan of care.

**Table 1:** The patient's plan of care.

Sr.no.	Date of events	Consultation	Events
1.	24/11/2020	Oncology department	Diagnosis of left Metaplastic breast carcinoma
2.	28/12/2020	Medical department	Diagnosis of pleural effusion
3.	21/4/2021	Medical department	Thoracocentesis on the right side
4.	5/11/2021	Medical department	Hospital admission
5.	6/11/2021	Medical ICU	HRCT Scan of the thorax
6.	7/11/2021	Medical ICU	Physiotherapy referral
7.	4/12/2021	Medical ward	Discharge
8.	18/12/2021	Physiotherapy outpatient department	Follow-up

Initial observation revealed that the patient was fine, although she was lethargic during the clinical examination. On inspection, pallor was present. The inspiratory-to-expiratory (I: E) ratio was evident of shallow breathing. In addition, there was reduced chest expansion at supramammary, mammary, and inframammary levels. Percussion revealed stony dullness in both lower zones with reduced air entry bilaterally on auscultation. Table 2 shows the pulmonary rehabilitation intervention.

**Table 2:** Pulmonary rehabilitation intervention PLB: pursed lip breathing; PMR; progressive muscle relaxation; ACBT; active cycle of breathing technique; PT: physiotherapy

S.no	Goals	PT intervention	Description of the intervention	
1.	To reduce anxiety and depression.	Guidance for the patient's caregivers.	Individual instruction on the significance of pulmonary rehabilitation, palliative care, and adherence to an exercise program.	
2.	To relieve breathlessness.	Side-lying chest to knee and forward-leaning when sitting are two postures that can help with dyspnea, as can doing PLB while in these positions.	Every time the patient becomes dyspneic, she is asked to perform these exercises.	
3.	To induce relaxation.	Positional changes and general relaxation include providing proper support and comfort to the body using pillows.	Every two hours.	
		PMR was achieved using Jacobson’s technique of relaxation.	Two to three sessions per day for five to 10 minutes.	
4.	To promote airway clearance and maximize secretion removal.	ACBT was recommended after bronchodilator nebulization.	Three to four rounds of ACBT after each PT session.	
5.	To improve the ventilation and oxygenation of the lung.	Exercises for segmental breathing, such as apical and lateral costal expansion, diaphragmatic breathing, and PLB, are also recommended.	Two to three sessions per day for five to 10 minutes.	
		Breath stacking exercise	Five repetitions, three times a day.	
		Incentive spirometer exercise in an upright position as shown in Figure 2		

**Figure 2:**
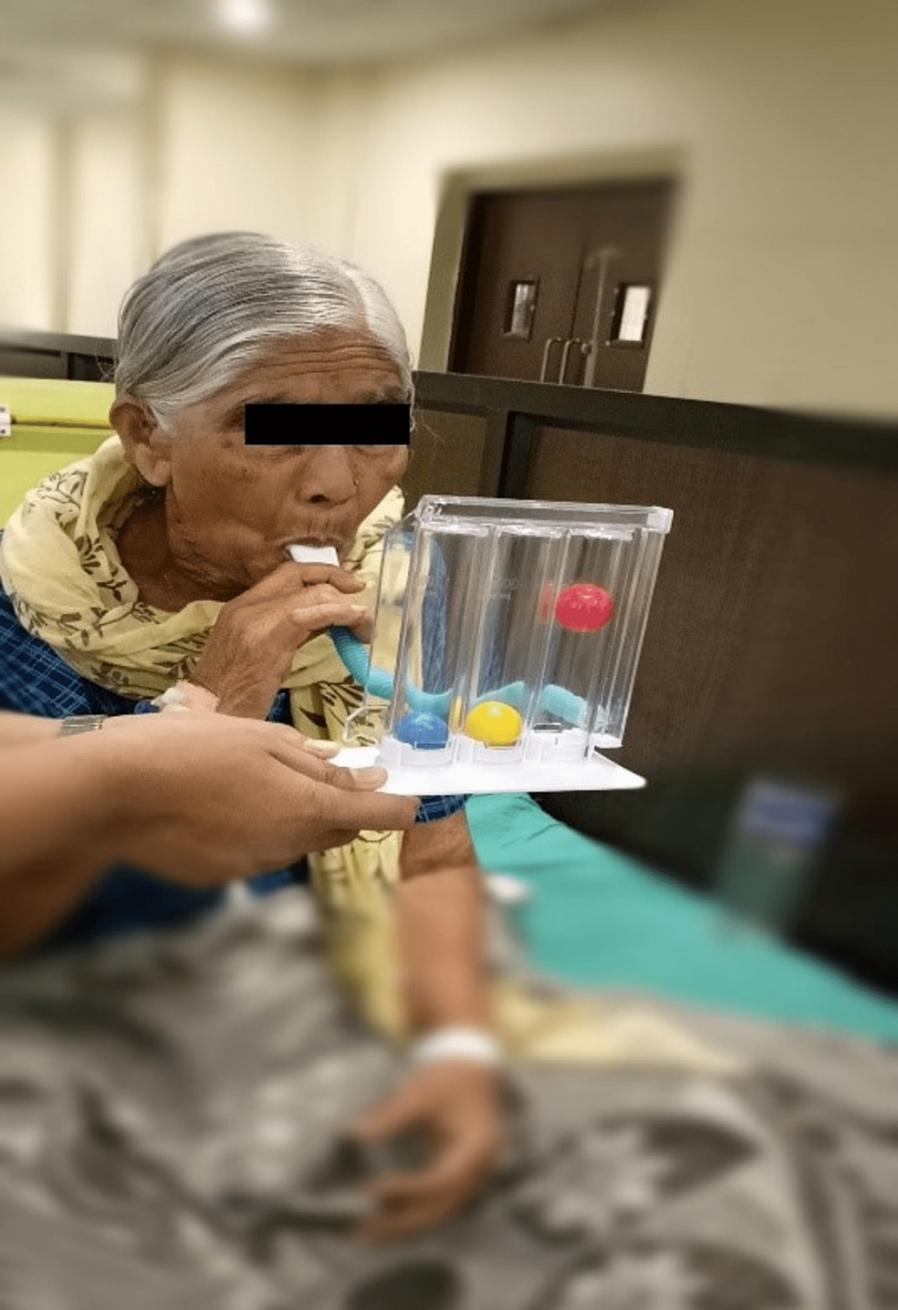
Patient performing incentive spirometry in an upright sitting position.

On discharge, the patient followed a defined two-week home-exercise routine and a follow-up, and the patient kept in touch telephonically for protocol adherence. Her exercise routine consisted of resistance training, aerobic training, stretching exercises, and pacing techniques. The American College of Sports Medicine (ACSM) recommends doing main muscle group exercises twice a week for two days, hence that was recommended. Walking was chosen as a mode of aerobic training for the remaining four days.

The FACIT-F (Functional Assessment of Chronic Illness Therapy-Fatigue) scale was used to determine the fatigue level and was acclimated from the Modified Medical Research Council scale to determine the dyspnoea grade (mMRC), using the Hospital Anxiety and Depression Scale (HADS). It uses the Karnofsky Performance Status Rating scale to assess anxiety and depression, as well as the physical workouts shown in Table 3. There were whopping improvements seen in every order.

**Table 3:** The values of outcome measures used to evaluate the improvement of the patient.

Outcomes	Day 1	Week 2	Discharge	Follow-up
Functional assessment of chronic illness therapy -Fatigue	8	34	40	48
Hospital anxiety scale	Anxiety (19)	Anxiety (13)	Anxiety (7)	Anxiety (5)
Depression scale	Depression (17)	Depression (11)	Depression (8)	Depression (4)
Oxygen therapy	Face mask providing 10 L O2 per minute.	Nasal prongs at a rate of 2 L O2 per minute	Keeping the air in the room saturated.	Controlling the air in the room drenched.
Grade of dyspnoea (modified Medical Research Council scale)	III	II	I	I
Karnofsky performance status rating	20	50	70	80

## Discussion

Palliative care is the most effective therapy for cancer patients. It aids in the improvement of the patient's and their family's quality of life [8]. Anesthetic care focuses on symptoms that arise from cancer and can be managed with both pharmacological and non-pharmacological approaches [9]. The primary interventions for rehabilitating patients with deteriorating health status due to respiratory problems include patient education, airway clearance procedures, breathing strategies, and a graded exercise training program [10]. Deep breathing, incentive spirometry, and positive pressure exercises were all effective lung expansion techniques employed in patients who had drained pleural effusions [11]. Chest mobility exercises improve chest wall, trunk, and shoulder mobility, even focusing on depth of inspiration. These exercises can help people with pleural effusion improve their chest expansion. Tahir et al. [12] discovered that breathing in segments is more effective and beneficial than deep breathing exercises in improving chest expansion and lung function in pleural effusion patients [12].

The pulmonary rehabilitation program is a multidisciplinary team-based intervention. It entails the participation of a variety of specialists who are working toward a shared objective, and research has shown that working as a team improves outcomes [13]. Cancer rehabilitation is now a viable alternative for cancer survivors. It is a procedure that is significant at various points in the lives of cancer patients, beginning with the initiation of therapy [14]. In addition, cancer patients must receive palliative care for several reasons. The number of studies examining the advantages of biological exercise for palliative care patients has steadily increased. According to the findings of a systematic review, exercise as a preventive measure has shown improvement in aerobic fitness, strength, and physical function are all necessary, as well as pain and exhaustion [15]. According to estimates, cancer affects approximately one million people in India. Previously, analgesic care was believed to be a treatment that would result in a patient's complete recovery, but now the goal of palliative care is expected to enhance the patient's overall life satisfaction. Physiotherapy has been shown to improve perceived well-being in a variety of patient populations in need of palliative care, including cancer, HIV, neurological issues, cardiac issues, and mental illnesses [16].

## Conclusions

The intervention in this case study concentrated on palliative care. Physiotherapy rehabilitation has proven to be quite effective for individuals receiving palliative care. The therapist repeatedly advised the patient to pursue palliative care rehabilitation programs to aid with dyspnea, chest discomfort, and weakness. This case report conveys the management direction and importance of adherence to exercise in lung cancer patients in terms of pulmonary rehabilitation and palliative care physiotherapy to bring out the best possible physical and mental health. The therapy has resulted in a considerable improvement in the patient's quality of life in addition to a decrease in symptoms.
